# Estimated Costs Associated With Management of Otosclerosis With Hearing Aids vs Surgery in Europe

**DOI:** 10.1001/jamanetworkopen.2021.48932

**Published:** 2022-02-17

**Authors:** Sophie Bonnafous, Jennifer Margier, Sophie Bartier, Romain Tournegros, Stéphane Tringali, Maxime Fieux

**Affiliations:** 1 Hospices Civils de Lyon, Centre Hospitalier Lyon Sud, Service d’oto-rhino-laryngologie, d’otoneurochirurgie et de chirurgie cervico-faciale, Lyon, France; 2 Hospices Civils de Lyon, Pôle de Santé Publique, Service d’ Évaluation Économique en Santé, Research on Healthcare Performance (RESHAPE), INSERM U1290, Université Claude Bernard Lyon 1, Lyon, France; 3 Service d’oto-rhino-laryngologie, de chirurgie cervico faciale, Hôpital Henri Mondor, Assistance Publique des Hôpitaux de Paris, Créteil, France; 4 Univ Paris Est Creteil, INSERM, Institut Mondor de Recherche Biomédicale, Créteil, France; 5 Centre National de la Recherche Scientifique, Equipe Mixte de Recherche 7000, Créteil, France; 6 Université de Lyon, Université Lyon 1, Lyon, France

## Abstract

**Importance:**

Surgery and hearing aids have similar outcomes in terms of hearing acuity but differ in terms of cost, aesthetics, and patient quality of life. The cost-effectiveness and budget impact of otosclerosis treatments have never been studied in Europe.

**Objectives:**

To compare the estimated mean costs per patient over 10 years of surgery vs hearing aids for the treatment of otosclerosis and to estimate the budget impact of an increase in the proportion of patients receiving surgical treatment.

**Design, Setting, and Participants:**

This economic evaluation analyzed French and European epidemiological data on the surgical management of symptomatic otosclerosis and compared them with data from the literature to build economic models. The analysis was conducted in January 2021.

**Exposures:**

Two care pathways were considered in the treatment of otosclerosis, either hearing aid or surgery.

**Main Outcomes and Measures:**

Costs were studied over 10 years using Markov models of the 2 care pathways (hearing aid vs surgery). The budget impact analysis was performed over 5 and 10 years, assuming a 1–percentage point yearly increase in the proportion of patients receiving surgical treatment.

**Results:**

Over 10 years, the estimated mean cost per patient was significantly lower in the surgery group compared with the hearing aid group (€3446.9 vs €6088.4; mean difference, −€2641.5; 95% CI −€4064.8 to −€1379.4 [US $3913.4 vs US $6912.4; mean difference, −US $2999.0; 95% CI, −US $4614.9 to −US $1566.1]). Increasing surgical treatment by 1 percentage point per year for 10 years would lead to overall savings of €1 762 304 (US $2 000 798) in France, with an increase of €1 322 920 (US $1 501 952) at 10 years for the public health insurance system and a decrease of €3 085 224 (US $3 502 750) at 10 years for patients and private health insurers. Sensitivity analyses showed that these results were robust.

**Conclusions and Relevance:**

These results suggest that in France, treating otosclerosis surgically is slightly less expensive over 10 years than using hearing aids, when considering all payers. The proposed models developed in this study could be adjusted to perform the same analysis in other countries.

## Key Points

**Question:**

What is the cost of otosclerosis, and what is the potential budget impact of an increase in the proportion of patients receiving surgical treatment?

**Findings:**

In this economic evaluation, the mean cost of surgical treatment per patient was substantially lower than using hearing aids (approximately €2000 lower) even if all patients with hearing aids were to choose the most inexpensive devices. Similarly, increasing the incidence of surgical treatment for otosclerosis was associated with reduced overall cost of treatment.

**Meaning:**

In this study, the mean cost of surgery per patient for otosclerosis was found to be lower than treatment with conventional hearing aids no matter the price of devices, and increasing the incidence of surgical treatment was cost-effective.

## Teaser

This economic evaluation compares the estimated mean costs per patient over 10 years of surgery vs hearing aids for the treatment of otosclerosis and estimates the budget impact of an increase in the proportion of patients receiving surgical treatment.

## Introduction

Otosclerosis is a condition of multifactorial origin caused by abnormal bone remodeling in the middle ear. Its incidence has been estimated between 3.9 per 100 000 individuals and 1 per 100 individuals,^1,2^ with female predominance. Although its incidence seems to be decreasing,^1,3^ otosclerosis remains the main cause of conductive hearing loss with intact tympanic membrane. In mild cases, treatment can involve monitoring, but patients with an air-bone gap greater than 20 dB are typically offered hearing aids or surgical treatment.

Regardless of the technique used, operations can safely be performed as outpatient procedures.^4^ The success rate is greater than 90%, and long term outcomes (>5 years) are good (residual air-bone gap lower than 10 dB)^1,5,6,7,8,9,10,11^ and associated with a very low (ie, <1%) risk of deafness.^5,6,7^ However, other complications need to be considered, such as (1) revision surgery because of decreased hearing, complications, or lack of improvement after the initial operation and (2) use of a hearing aid after surgery because of decreased hearing or insufficient improvement.^8,9,10,11^ The risks related to surgery are therefore greater than those related to hearing aids and also have financial consequences.

Adverse effects from hearing aid use are common but mild. Otitis externa and impacted cerumen are twice and four times, respectively, as common among hearing-aid users as in the general population,^12^ and consultations for eczema of the ear canal and foreign objects trapped in the ear are also more frequent.^12^ These problems add to the costs of hearing aids themselves, which can be high and are often not covered by health insurance. Prices vary widely between devices. In France, class I hearing aids (first level hearing aid) have a fixed maximum price of €950 and are fully reimbursed (no out-of-pocket costs), but patients choosing class II hearing aids (freely priced) have to pay most of the cost themselves (only part is reimbursed).^13^

Overall, surgery and hearing aids have similarly good outcomes in terms of hearing acuity but differ in terms of cost, aesthetics, and patient quality of life.^6,7,14,15^ Given that there are very few contraindications for surgery (single ear and gusher syndrome), the choice of treatment is left to patients based on the benefits and risks of the 2 approaches.^5^ However, the costs for public health insurance systems, patients, and private health insurers vary between treatment modalities and countries.

In a recent US study comparing the cost-effectiveness of surgery and hearing aids for otosclerosis,^11^ the incremental cost-effectiveness ratio of stapedectomy was found to be $3918.43 per quality-adjusted life year (QALY). Based on the commonly used threshold of $50 000 per QALY, surgery was therefore deemed to be cost-effective compared with hearing aid use. This result can be explained by the young age of patients with otosclerosis at presentation, the effectiveness of surgery, and its impact on quality of life. To our knowledge, the cost-effectiveness and budget impact of otosclerosis treatments have never been studied in France or elsewhere in Europe. The importance of quantifying the economic impact of health care interventions has been highlighted by the ongoing COVID-19 pandemic, which is directly responsible for a social and financial crisis.

The principal objective of the present study was to compare the mean overall costs of surgery and hearing aids for the treatment of otosclerosis. The secondary objective was to perform a budget impact analysis (BIA) at 5 and 10 years of a 1–percentage point yearly increase in the number of patients opting for surgery rather than hearing aids from the perspective of the public health insurance system as well as from the perspective of patients and private insurers.

## Methods

### Study Design

A systematic review of the literature was performed to implement the economic models and perform cost and budget impact analyses in January 2021. Surveys were also conducted in France and in Europe to determine the epidemiological characteristics of otosclerosis treatment. No patients were involved.

### Ethics Approval

This study complies with the ethical and legal requirements of the French law (April 15, 2019) and the Declaration of Helsinki.^16^ This study was approved by an institutional review board Comité d’Ethique du CHU de Lyon. This study followed the Consolidated Health Economic Evaluation Reporting Standards (CHEERS) reporting guideline.

### Epidemiological Analysis of the Treatment of Otosclerosis

**Figure 1.**
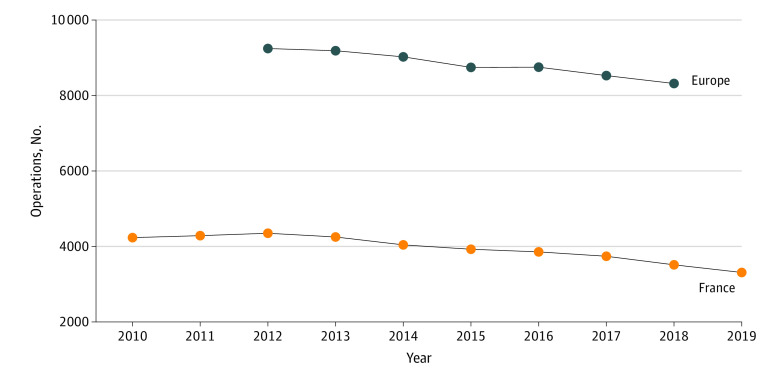
Annual Number of Operations Performed to Treat Otosclerosis in France and in a Group of European Countries Europe includes operations performed in Belgium, Luxembourg, Switzerland, Sweden, Germany, and Norway.

This analysis was based on the literature and retrospective data on hospital stays from the French Diagnosis-Related Group system (Programme de Médicalisation des Systèmes d’Information). Information was also gathered from public health databases in France and other European countries, namely Sweden,^17^ Switzerland,^18^ Germany,^19^ and Norway.^20^ Full details regarding the epidemiological analysis are provided in eAppendix 1 in the Supplement. The economic models were implemented using data from the literature to ensure the cost and budget impact analysis were realistic. Otosclerosis symptoms appear between age 30 and 50 years.^1,2,6,21^ Between 13% and 58% of cases are hereditary.^1,21^ Otosclerosis surgery has a reported success rate of more than 90%.^1,5,7^ The estimated rate of severe complications (deafness) was less than 1%.^5,7^ Reported reoperation rates vary from 2% within 13 years to 10.5% over 15 years.^6,7,8^ The proportion of major middle-ear surgeries performed as outpatient procedures ranged from 27% to 90.4%, depending on the year and treatment center considered.^4,22^ Otosclerosis was bilateral in 75% of cases but often asymmetric, with surgical treatment of both ears in 27% to 34% of cases within 15 to 20 years.^1,21,23,24,25^ The number of surgical procedures performed for the treatment of otosclerosis has been decreasing since 2012 by approximately 3.3% per year in France^26^ as well as in the other European countries for which data were available (Table 1, Figure 1).^27,28,29,30,31,32,33,34,35^ The proportion of patients using hearing aids within 10 years after surgery ranged from 29% to 54%.^7,9^ There was no information available in the literature regarding the proportion of patients receiving surgery or hearing aids as first-line treatment. In the United-States, 67% of patients diagnosed with otosclerosis are first treated surgically.^2^

**Table 1.** Number of Otosclerosis Operations Per Year and Estimated Incidence of Surgical Treatment For Otosclerosis^a^

Country	Total operations, No.										Incidence in 2012		Incidence in 2018		*P* value^b^
	2010	2011	2012	2013	2014	2015	2016	2017	2018	2019	No. per 100 000 population	Source	No. per 100 000 population	Source	
**France**	4236	4287	4354	4253	4044	3924	3874	3742	3515	3311	6.6	INSEE,^27^ 2013	5.3	INSEE,^28^ 2018	.79
**Switzerland**	316	10	253	289	321	309	306	358	319	398	3.2	Country Meters^29^	3.8	Country Meters^29^	>.99
**Luxembourg**	24	21	30	22	20	22	19	26	16	27	5.7	Population Pyramid^30^	2.5	Population Pyramid^30^	.51
**Sweden**	NA	NA	129	277	356	367	344	331	360	389	1.4	Country Meters^31^	3.6	Country Meters^31^	.69
**Belgium**	533	577	507	548	452	470	659	637	572	574	4.6	Population Pyramid^32^	5	Population Data^33^	>.99
**Germany**	4246	4013	3735	3624	3638	3464	3379	3263	3362	NA	4.6	Country Meters^34^	4.1	Country Meters^34^	>.99
**Norway**	200	204	243	177	195	192	174	171	178	214	4.8	Country Meters^35^	3.3	Country Meters^35^	>.99
**Total**	NA	NA	9251	9190	9026	8748	8755	8528	8322	NA	NA	NA	NA	NA	NA

Abbreviation: NA, not applicable.

^a^ The total corresponds to the sum of cases in France, Switzerland, Luxembourg, Sweden, Belgium, Germany, and Norway. The incidences correspond to the number of cases in a given year divided by the population of the corresponding country during that year.

^b^ Fisher exact test. Significant if *P* < .05.

The incidence of symptomatic otosclerosis was very difficult to evaluate; rates between 1 to 3.9 per 100 000 individuals and 1 per 100 individuals^1,2^ have been reported, and it has been decreasing. Indeed, in the United States, the incidence has decreased from 18.5 per 100 000 person-years in the 1970s (at the beginnings of stapes surgery) to 3.9 per 100 000 person-years between 2015 and 2017. Based on the reported incidence among White individuals in the United States and the decreasing trend over time, the incidence in France in 2017 was estimated to be 5.2 per 100 000 person-years.^2^

### Statistical Analysis

#### Treatment Cost Analysis

**Figure 2.**
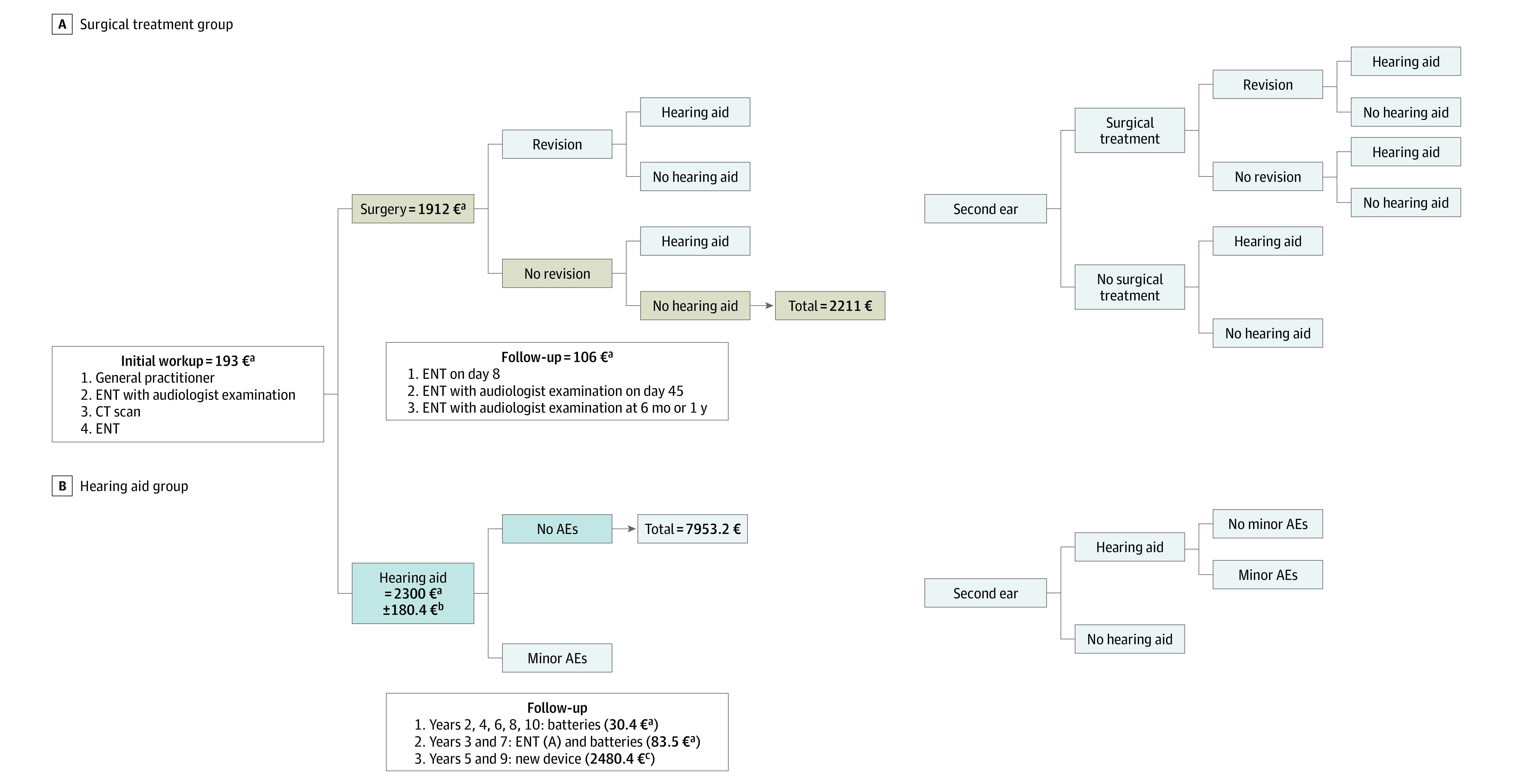
Care Pathway and Costs for Surgery and Hearing Aids In each 1-year step, patients (first ear) are in 1 of 4 states in the surgery group and in a single (absorbing) state in the hearing aid group. Each state has an associated cost (surgery group, cost of surgery without hearing aid or revision surgery during the follow-up; hearing aid group, cost of class III hearing aid treatment over 10 years). The estimated annual probabilities of revision surgery and postoperative hearing aid use are 0.004 and 0.013, respectively.^4^ The estimated annual probability of requiring treatment in the other ear is 0.02.^4^ Patients in the hearing aid group can have minor adverse effects (AEs) related to hearing aid use, whose estimated annual probability is 0.24,^12^ and they can also require treatment in the other ear, with an estimated annual probability of 0.02.^4^ Annual transition probabilities were estimated from a local survey and literature data. The shaded chains correspond to unilateral outpatient surgery (Diagnosis-Related Group 03C20J) treatment over 10 years with no revision surgery or postoperative hearing aid use and class III hearing aid treatment over 10 years. CT indicates computed tomography; ENT, ear, nose, and throat. To convert euros to US dollars, multiply by 1.14. ^a^Overall cost per patient (covered by public health insurance and paid by patients or private health insurers). ^b^Batteries and charge for hearing aid. ^c^Class III device with batteries and charge.

The objective was to calculate the mean cost per patient depending on whether they required surgery (with or without hearing aids) or solely hearing aids for the treatment of otosclerosis. Cost analyses were divided between individuals, private health insurers, and the public health insurance system according to current arrangements in France, where surgery is mainly covered by public health insurance, but hearing aids are often paid for in part by individuals and private health insurers. Cost values were expressed in euros (to convert to US dollars, multiply by 1.14). The time period considered for each patient was 10 years, which corresponds to the lifetime of 2 hearing aids and is also long enough for contralateral symptoms and events associated with postoperative complications (revision surgery, postoperative hearing aid use) to occur. The targeted population was patients with symptomatic otosclerosis in need of active treatment (hearing aids or surgery). They were attributed to the surgery group (if they underwent surgery) or to the hearing aid group (if they did not undergo surgery) (Figure 2). Costs were calculated for care pathways established based on the literature and current practice after multidisciplinary discussions with an ear, nose, and throat (ENT) specialist, a radiologist, audiologists, and a public health physician (eTable 1 and eTable 2 in the Supplement). A Markov decision process was used to illustrate patient care pathway (Figure 2) and determine the mean cost of the 2 initial treatments based on the expected distribution of outcomes. Further details are provided in eAppendix 2 in the Supplement.

The parameters of the model, derived from the epidemiological analysis, are presented in Table 2.^2,4,7,8,12,26,36^ For the hearing aid group, costs were determined on a yearly basis, with a new device every 4 years (Table 2). Based on audiologists’ estimates and sales figures provided by the Syndicat National de l’Industrie des Technologies Médicale, we assumed that 20% of patients using a hearing device would choose a class I device (the minimum target set by the state for audiologists in France), 35% would choose a class II, subgroup C or D device (class II), and 45% would choose a class II, subgroup E or F device (class III). In years 2, 4, 6, 8, and 10 following hearing device choice, the only costs related to hearing device considered were those of batteries. The cost of an ENT consultation was included in years 3 and 7, and the cost of a new hearing aid was added in years 5 and 9. Indeed, according to the survey of audiologists we conducted, the health insurance system reimburses its share of hearing aids every 4 years, allowing patients to update their hearing aids. The hospitalization costs associated with initial and revision surgery were obtained from the 2018 French survey of health care costs (Étude Nationale des Coûts; the last available data set). The distribution in terms of Diagnosis-Related Groups was obtained from the ScanSanté website for 2019 (Table 1).^26^ The costs of consultations and of medical examinations were set to the current regulated fees in France (eTable 1 and eTable 2 in the Supplement). The costs of hearing aids and hearing aid batteries were estimated from a survey of audiologists and the literature. Costs were discounted at 2.5% per year as recommended.^37^ Deterministic (tornado diagram) and probabilistic sensitivity analyses were performed to test the robustness of the results. For the probabilistic analysis, costs were assumed to follow γ distributions and probabilities β distributions. Bootstrapping with 1000 samples was used to simulate a 95% CI for the difference in costs.^38^ All model variables were included in the analysis.

**Table 2.** Parameters Considered in the Budget Impact Analysis Model

Parameter	Value, %	Source
Change in the incidence of otosclerosis		
Baseline estimate	−3.3	Marinelli et al,^2^ 2020
Annual change in the proportion of patients receiving surgical treatment		
Baseline estimate	1 percentage point per year on average	2 percentage points (year 1), 1.5 percentage points (year 2), 1 percentage points (year 3), 0.5 percentage points (year 4), and 0.5 percentage points (year 5)
Distribution of initial surgeries by DRG code		
03C20J	34	ScanSanté,^26^ 2019
03C201	64	
03C202	2	
Distribution of revision surgeries by DRG code		
03C16J	25	ScanSanté,^26^ 2019
03C161	45	
03C162	30	
Distribution of hearing aids by class		
Class I	20	Sales statistics for 2019 in France (SNITEM)
Class II	35	
Class III	45	
Upper estimate	100% class I	
Reoperation rate		
Baseline estimate	9.4	35 of 373 patients, ie, 0.4% per patient per year (Bonnafous et al,^4^ 2020)
Upper estimate	10.5	Bakhos et al,^8^ 2010
Lower estimate	6	ScanSanté,^26^ 2019
Proportion of patients treated with hearing aids		
Baseline estimate	15	
Upper estimate	33	Marinelli et al,^2^ 2020
Lower estimate	6.7	8 of 119 patients had no surgery (Bonnafous et al,^4^ 2020)
Risk of postoperative hearing aid use		
Baseline estimate	13.6	35 of 373 patients, ie, 1.3% per patient per year over 7 years (Bonnafous et al,^4^ 2020)
Upper estimate	54	Lucidi et al,^7^ 2020
Lower estimate	0	
Risk of bilateral otosclerosis		
Baseline estimate	9	34 of 373 patients, ie, 2% per patient per year (Bonnafous et al,^4^ 2020)
Upper estimate	53	Lucidi et al,^7^ 2020
Lower estimate	6	Rajput et al,^36^ 2020
Risk of minor hearing aid side-effects		
Baseline estimate	24% per year	Seidel et al,^12^ 2019
Hearing aid cost		
Class 1	€950	Regulated price
Class 2	€1500	Survey of audiologists
Class 3	€2300	Survey of audiologists
Hearing aids minor adverse events cost	€53.00	2020 Tariff
Surgery cost		
03C20J	€1892.07	2020 DRG fees
03C201	€1887.61	
03C202	€3124.03	

Abbreviations: 03C20J, outpatient middle ear surgery; 03C201, middle ear surgery, level 1; 03C202, middle ear surgery, level 2; 03C16J, other outpatient ear, nose, throat, or neck surgeries; 03C161, other outpatient ear, nose, throat, or neck surgeries, level 1; 03C162, other outpatient ear, nose, throat, or neck surgeries, level 2; DRG, Diagnosis-Related Group; SNITEM, Syndicat National de l’Industrie des Technologies Médicale.

#### BIA at 5 and 10 Years

**Figure 3.**
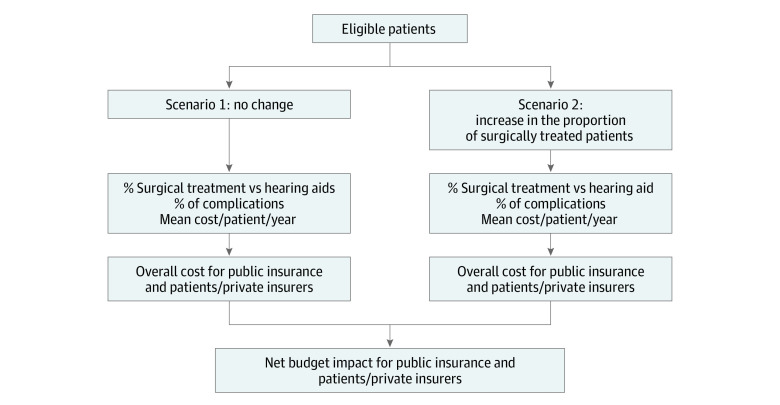
Schematic Representation of the Budget Impact Analysis Model

The aim of the BIA was to estimate the financial impact of an increase in the proportion of patients receiving surgical treatment. We compared a continuation of the current situation as a baseline scenario (scenario 1) with an alternative scenario (scenario 2) in which a greater proportion of patients would choose surgery rather than hearing aids (Figure 3). Contrary to guidelines recommending a single perspective,^39^ we considered costs from 3 points of view, namely public health insurance, patients, and private health insurers, and overall. The costs of hearing aids are indeed often covered in large part by private health insurers and patients themselves, and this perspective is more internationally relevant. The targeted population was once again patients with symptomatic otosclerosis in need of active treatment (hearing aids or surgery).

The BIA was modeled over 5 and 10 years using a multicohort approach. In the first year, only incident cases were considered, whereas in subsequent years, new cases were added to those already included. The distribution of patients between the 2 treatment options in scenario 1, based on current estimates, was 85% for surgery and 15% for hearing aids. In scenario 2, based on expert opinion, the proportion of patients opting for surgery increased from baseline by 1 percentage point per year. The patients’ care pathways (eTable 1 and eTable 2 in the Supplement) and the parameters of the model (Table 1) were the same as those for the cost analysis. Treatment costs were divided between the public health insurance system and patients or private health insurers, as described previously for the cost analysis. Excess hospitalization fees (greater than state-regulated prices) in private hospitals were not considered. Indirect costs (per sick leave) were excluded from the reference case analysis but included in the sensitivity analysis as recommended in the methodological guidance.^40^ Deterministic sensitivity analysis including all model parameters was performed to test the robustness of the 10-year results. Budget impact analyses were performed using Excel. Statistical analyses were performed using R version 4.0.4 (R Project for Statistical Computing). Results were considered statistically significant at *P* < .05, and all tests were 2-tailed.

## Results

Over 10 years, the estimated mean cost per patients was significantly lower in the surgery group compared with the hearing aid group (€3446.9 vs €6088.4; mean difference, −€2641.5; 95% CI −€4064.8 to −€1379.4 [US $3913.4 vs US $6912.4; mean difference, US −$2999.0; 95% CI, −US $4614.9 to −US $1566.1]). Sensitivity analyses (eFigure and eTable 3 in the Supplement) identified the following contingencies. The annual probability of revision surgery had to be at least 10% for the cost difference to favor hearing aids (+€123 [US $140] for a probability of 10% per year). The annual probability of postoperative hearing aid use had to be at least 14% for the cost difference to favor hearing aids (+€61 [US $69] for a probability of 14% per year). For patients older than 70 years, surgery is sometimes performed simply to ensure hearing aids are effective; therefore for these patients, the cost over 10 years after a surgical treatment for the fitting of a hearing aid is €8918 (US $10 125) (vs €3347 [US $3800] for a patient treated with surgery and €6088 [US $6912] for a patient treated with hearing aids alone). Increasing the proportion of outpatient surgeries (initial and revision) to 100% (currently 34% for initial and 25% for revision surgery) would not substantially reduce costs for patients (−€94 [US $107] for the initial surgery; and −€15 [US $17] for revision surgery). The cost difference remained in favor of surgery even though all patients were assumed to use class I (€950 [US $1079]) hearing aids (cost differences: for 100% of patients using class I hearing aids, −€429 [US $487]; for 20% of patients using class I hearing aids, −€2641 [US $2998]). Assuming 50% of patients would require treatment for their other ear within a year, the cost difference became more strongly in favor of surgery (−€4855 [US $5512]). Surgery fees would have to be increased by 91% (ie, to €3724 [US $4228] for outpatient middle ear surgery) to bring the cost difference to nearly zero (−€1 [US $1]). The cost per patient in the surgery group depended strongly on the length of sick leave after surgery. The cost difference remained in favor of surgery (−€1440 [US $1635]) if the postoperative sick leave was assumed to last 7 days but moved toward hearing aids at 16 days of postoperative sick pay (+€105 [US $119]).

In the baseline BIA scenario (scenario 1), the cost of symptomatic otosclerosis in France was €26 531 426 (US $30 121 950) over 5 years and €54 194 976 (US $61 529 236) over 10 years for the public health insurance system and €12 625 061 (US $14 333 623) over 5 years and €31 922 580 (US $36 242 695) over 10 years for patients and private health insurers; hence, the overall cost was €39 156 487 (US $44 455 574) over 5 years and €86 117 557 (US $97 771 932) over 10 years.

In the alternative scenario (scenario 2), the cost of symptomatic otosclerosis in France was €27 287 107 (US $30 979 898) over 5 years and €55 517 896 (US $63 031 188) over 10 years for the public health insurance system and €11 822 179 (US $13 422 086) over 5 years and €28 837 356 (US$32 739 944) over 10 years for patients and private health insurers; hence the overall cost was €39 109 286 (US $44 401 985) over 5 years and €84 355 252 (US $95 771 133) over 10 years. The estimated net incremental budget impact (scenario 2 minus scenario 1) was €755 681 (US $857 948) at 5 years and €1 322 920 (US $1 501 952) at 10 years for the public health insurance system (in favor of scenario 1), −€802 882 (US $911 536) at 5 years and −€3 085 224 (US $3 502 750) at 10 years for patients and private health insurers (in favor of scenario 2), and −€47 200 (US $53 587) at 5 years and −€1 762 304 (US $2 000 798) at 10 years overall (in favor of scenario 2). The overall estimated net budget impact at 4 years (before hearing aid replacement) was €62 529 (US $70 991).

Sensitivity analyses were performed, and full details are provided in eAppendix 3 in the Supplement. The annual probability of revision surgery had to be at least 10% for the overall incremental budget impact at 10 years to favor scenario 1 (€62 965 [US $71 486] for a probability of 10% per year), although the budget impact remained in favor of scenario 2 (−€3 017 472 [US $3 425 830]) for patients and private health insurers. Regarding the length of postoperative sick leave, the estimated overall budget impact was in favor of scenario 1 at 30 days (€16 350 [US $18 563] at 31 days).

## Discussion

The incidence of surgical treatment for otosclerosis has been decreasing in several European countries even though the mean cost of surgery per patient was found to be lower than treatment with conventional hearing aids. Similarly, in France, increasing the incidence of surgical treatment for otosclerosis would slightly increase costs from the public health insurance system perspective but could significantly reduce the cost for patients and private insurance. Collectively, the overall cost of treatment could be cost-saving, but it would be at the expense of the public health insurance system.

In terms of mean costs per patient over 10 years, surgery was found to be roughly €2641.5 (US $2999.0) less expensive than hearing aids, which is in disagreement with the recent cost-effectiveness study by Gillard et al,^11^ which found that the lifetime incremental cost of surgery was US $2978.01 higher, although surgery was still found to be cost-effective.^11^ This difference can be explained by the fact that surgery is actually 2.5 times more expensive in the United States than in France ($5394), whereas the cost of hearing aids ($2350) is similar. Furthermore, the substantial variation among devices^41^ contributes to the high variability of otosclerosis-related health care costs in the United States, which seems harder to estimate than in France. If the probabilities of revision surgery and postoperative hearing aid use were greater than 10%, the cost difference would favor hearing aids (€123 [US 140] for a 10% probability of revision surgery and €61 [US $69] for a 14% probability of postoperative hearing aid use). However, these values do not seem realistic based on our estimates and the literature. The length of postoperative sick leave was found to have a strong association with costs at the individual level in the surgery group.

The estimated overall incremental net budget impact was −€47 200 (−US $53 587) at 5 years and −€1 762 304 (−US $2 000 798) at 10 years (ie, in favor of the alternative scenario), indicating that increasing the proportion of patients treated surgically by 1 percentage point per year would reduce the overall cost of symptomatic otosclerosis treatment. The model only yielded the opposite result if certain variables were increased substantially compared with the baseline specification, namely the probability of revision surgery (up to 10% per patient per year), the probability of postoperative hearing aid use (up to 12% per patient per year), surgery fees (increased 60%), or the proportion of patients choosing class I devices (up to 94%). However, such values seem far from current realistic data.^4,7,8^ The increase in the proportion of outpatient procedures reduced public health insurance costs by more than €1 000 000 over 10 years despite a minute difference in costs between conventional and outpatient operations (€1912 [US $2171] vs €1928 [US $2189]).

The main strength of this study was the completeness of the epidemiological survey used to build the economic models, based an exhaustive review of the literature and of European health care databases. Sensitivity analyses were also performed and confirmed the robustness of the conclusions..

### Limitations

This study has limitations. Statistical models are by definition simplifications of the real world, and their objective herein was to grasp the financial complexity underlying the surgical treatment of patients with symptomatic otosclerosis. They did not account for subgroups with different clinical courses that may alter overall costs. For example, for patients older than 75 years, who are more likely to need hearing aids in addition to surgery, individual and overall costs may be greater. Nevertheless, our results are consistent with other countries. Indeed, stapes surgery was proven to be economically beneficial in Germany for the individual patient as well as for the general patient cohort, while they were hospitalized for 3 to 5 days and the basic hearing aid (also reimbursed) is less expensive than the one in France.^42^ Therefore, our model could be adapted for use in other countries. We have assumed that the results and mean cost of additional patients receiving surgery would be the same as those currently receiving surgery. It might possible that this specific population slightly differs in terms of cost and consequence. Unfortunately, it was not possible to obtain a better estimation for this specific population because data were missing. However, numerous sensitivity analyses have been performed and seemed to confirm the robustness of our results.

## Conclusions

The incidence of surgical treatment of symptomatic otosclerosis has been decreasing in a number of countries in Europe. However, the modeling of the health care system in France presented in this study indicates that increasing the incidence of surgical treatment by 1 percentage point per year would lead to savings of €1 762 304 (US $2 000 798) over 10 years. The proposed models could be adapted for use in other countries.

## Notes

Supplement. **eAppendix 1. **Epidemiological Analysis **eAppendix 2.** Treatment Cost Analysis, the Markov Model **eAppendix 3. **Supplementary Results: Sensitivity Analysis **eTable 1.** Clinical Course and Associated Costs of Patients in the Surgery Group **eTable 2*.*** Clinical Course and Associated Costs of Patients in the Hearing Aid Group **eFigure*.*** Tornado Diagram for Mean Cost per Patient **eTable 3***.* Lower and Upper Estimates for the Variables Considered in the Sensitivity Analysis
