# Supplementary material for: Estimated Costs Associated With Management of Otosclerosis With Hearing Aids vs Surgery in Europe
Source: JAMA Netw Open. 2022 Feb 17;5(2):e2148932. doi: 10.1001/jamanetworkopen.2021.48932 (PMC8855228; doi:10.1001/jamanetworkopen.2021.48932)
Supplement: Supplement. — eAppendix 1. Epidemiological Analysis eAppendix 2. Treatment Cost Analysis, the Markov Model eAppendix 3. Supplementary Results: Sensitivity Analysis eTable 1. Clinical Course and Associated Costs of Patients in the Surgery Group eTable 2. Clinical Course and Associated Costs of Patients in the Hearing Aid Group eFigure. Tornado Diagram for Mean Cost per Patient eTable 3. Lower and Upper Estimates for the Variables Considered in the Sensitivity Analysis [file jamanetwopen-e2148932-s001.pdf]

Supplemental Online Content

Bonnafous S, Margier J, Bartier S, Tournegros R, Tringali S, Fieux M. Estimated costs associated with management of otosclerosis with hearing aids vs surgery in Europe. *JAMA Netw Open.* 2022;5(2):e2148932. doi:10.1001/jamanetworkopen.2021.48932

eAppendix 1. Epidemiological Analysis

eAppendix 2. Treatment Cost Analysis, the Markov Model

eAppendix 3. Supplementary Results: Sensitivity Analysis

eTable 1. Clinical Course and Associated Costs of Patients in the Surgery Group

eTable 2. Clinical Course and Associated Costs of Patients in the Hearing Aid Group

eFigure. Tornado Diagram for Mean Cost per Patient

eTable 3. Lower and Upper Estimates for the Variables Considered in the Sensitivity Analysis

This supplemental material has been provided by the authors to give readers additional information about their work.

© 2022 Bonnafous S et al. *JAMA Network Open.*

eAppendix 1. Epidemiological Analysis

For Belgium, Luxembourg, Italy, Spain and Portugal public health or statistical agencies were contacted directly as the information was not available online. Only those from Belgium (Portahealth) and Luxembourg (the national social security website) responded. No source was found for other European countries. For France, the publicly accessible "ScanSanté" database provides hospitalisation data for all private and public healthcare centres. We counted the number of entries coded CBPA002 (stapedectomy and non-laser stapedotomy), CBPA003 (laser stapedotomy). and CBKA001 (replacement or repositioning of the stapes prosthesis after stapedectomy or stapedotomy). To estimate the incidence of otosclerosis surgery, we divided the number of patients who underwent otosclerosis surgery in 2012 and 2018 in a given country by the total population of this country during that year (obtained from national census websites). These two dates were chosen as they corresponded to the largest interval for which the necessary information was available in several European countries. Several other countries have publicly accessible records.

© 2022 Bonnafous S et al. *JAMA Network Open.*

eAppendix 2. Treatment Cost Analysis, the Markov Model

The Markov model is an analytical framework frequently used in decision analysis that accounts for events over time. Markov models use disease states to represent all possible consequences of an intervention of interest. At each time period called "cycle", a cohort of patients moves between the disease states according different probabilities. At each disease state, a cost and health outcome are associated. Finally, these results are aggregated over successive cycles to provide the total expected cost and outcome which can be compared with the aggregate results of a similar cohort receiving another intervention.

© 2022 Bonnafous S et al. *JAMA Network Open.*

eAppendix 3. Supplementary Results: Sensitivity Analysis

The annual probability of revision surgery had to be ≥ 10% for the overall incremental budget impact at 10 years to favour S1 (+62,965 € for a probability of 10% per year), although the budget impact remained in favour of S2 (−3,017,472 €) for patients and private health insurers. If the initial proportion of patients treated surgically was set to 50% (instead of 85%), the overall incremental budget impact at 10 years was −2,995,917 € in favour of S2. If the initial proportion of patients treated surgically was set to 95%, the overall incremental budget impact at 10 years was −1,532,726 €. The annual probability of postoperative hearing aid use has to be at least 0.12 for the overall incremental budget impact at 10 years to be in favour of S1 (+ 22,784 € at a probability of 12%) although the budget impact remains in favour of S2 (−2,887,251 €) for patients and private health insurers. Assuming that all surgical patients required hearing aids postoperatively, the overall cost of symptomatic otosclerosis was 185,191,587 € over 10 years in S1 and 188,563,802 € over 10 years in S2, corresponding to an overall budget impact of +3,372,215 €, i.e. in favour of S1 from the point of view of both the public health insurance system and patients and private health insurers. If all initial and revision surgeries were assumed to be performed as outpatient procedures, the overall cost in S1 was reduced by 1,181,476 € over 10 years (86,117,557 − 84,936,081 €) and by 1,246,453 € over 10 years in S2. If all patients were assumed to use class I hearing aids, the overall budget impact was in favour of S1 (+140,712 €), with no change from the perspective of the public health insurance system (since hearing aid reimbursements are already capped). If 94% of patients were assumed to use class I hearing aids, the overall budget impact was close to zero (−2,014 €). If the probability of bilateral surgery was assumed to be 0.5 per patient per year, the overall budget impact remained in favour of S2 (−3,053,398 €). Regarding the length of postoperative sick leave, the overall budget impact tips in favour of S1 at 30 days (+16,350 € at 31 days). Fees for initial surgery would have to be increased by more than 60% (i.e. up to 3027.3 € for outpatient middle-ear surgery) for the overall budget impact to favour S1 (+7,413 €). Assuming that all patients were to require follow-up appointments every year for mild hearing aid side-effects, the overall budget impact would remain in favour of S2 (−2,066,669 €). The overall budget impact remained in favour of S2 even if hearing aids were assumed to have no minor side effect (−1,684,301 €).

eTable 1. Clinical Course and associated Costs of Patients in the Surgery Group

Unilateral surgery	Regulated fee or retail price	Reimbursement rate	Amount covered by PuHI	Amount paid by patient or PrHI	Source: French DRG code or retail price
Pre-treatment workup					
General practitioner appointment	25.0 €	70%	16.5 €	8.5 €	C+MMG
ENT specialist consultation with audiology assessment	69.0 €	70%	47.3 €	21.7 €	APU
CT scan (basic fee + procedure-specific fee*)	26.0 €+ 48.0€	70%	64.9 €	8.8 €	LAQK002+YYYY600+FT
ENT consultation	25.0 €	70%	16.5 €	8.5 €	CS + MPC
Preoperative workup total	**193.0€**		145.2 €	**47.5 €**	
Surgery					
Outpatient middle-ear surgery (+ hospitalisation fee**)	**1 892 € (+20€)**	80%	**1 513.7 €**	**398.4 €**	03C20J
Middle-ear surgery, level 1 (+ hospitalisation fee**)	**1 888 € (+40€)**	80%	**1 510.1 €**	**417.5 €**	03C201
Middle-ear surgery, level 2 (+ hospitalisation fee**)	**3 124 € (+80€)**	80%	**2 499.2 €**	**704.8 €**	03C202
Sick pay	38.6 €/day			Basic allowance based on median 2018 income in France	
Postoperative follow-up					
ENT specialist consultation on day 8	0.0 €	100%	0.00 €	0.00 €	No charge
ENT specialist consultation with audiology assessment on day 45	53.1 €	70%	36.2 €	16.9 €	CDQP002
ENT specialist consultation with audiology assessment at 6 months or 1 year	53.1 €	70%	36.2 €	16.9 €	CDQP002
Postoperative follow-up total	**106.0€**		**72.2 €**	**33.9 €**	
Unilateral surgery TOTAL					
Outpatient middle-ear surgery	**2 211.0€**		**1 731.3 €**	**479.8 €**	
Middle-ear surgery, level 1	**2 138.9 €**		**1 727.7 €**	**498.9 €**	
Middle-ear surgery, level 2	**3 375.3 €**		**2 716.8 €**	**786.2 €**	

Legend: ** The procedure-specific fee is fully covered by PuHI.**The hospitalisation fee is paid by patients or their private health insurance.*
Abbreviations: *PuHI, public health insurance; PrHI, private health insurance; DRG, diagnosis-related group; ENT, ear, nose, and throat; CT, computerised tomography.*

© 2022 Bonnafous S et al. *JAMA Network Open.*

eTable 2. Clinical Course and Associated Costs of Patients in the Hearing Aid Group

Unilateral hearing aid	Regulated fee or retail price	Reimbursement rate or amount	Amount covered by PuHI	Amount paid by patient or PrHI	Source: French DRG code or retail price
Pretreatment workup					
General practitioner appointment	25.0 €	70%	16.5 €	8.5 €	C+MMG
ENT specialist consultation with audiology assessment	69.0 €	70%	47.3 €	21.7 €	APU
CT scan (basic fee + procedure-specific fee*)	26.0 € +48.0€	70%	64.9 €	8.8 €	LAQK002+YYYY600+FT
ENT specialist consultation	25.0 €	70%	16.5 €	8.5 €	CS + MPC
Preoperative workup total	**193.00€**		**145.2 €**	**48.2 €**	
Hearing aid					
Class I device	950.0 €	400€	400 €	550 €	Fixed by PuHI
Class II device	1 500.0 €	400€	**400 €**	**1 100 €**	Retail price*
Class III device	2 300.0 €	400€	400 €	**1 900 €**	Retail price*
Battery charger	150.0 €	0%	0 €	**150 €**	Retail price
Battery (p13)	30.4 €	30%	9 €	**21 €**	Retail price**
Hearing aid and workup total					
Class I device	**1323.4€**		**554 €**	**769 €**	
Class II device	**1873.4€**		**554 €**	**1 319 €**	
Class III device	**2673.4€**		**554 €**	**2 119 €**	
Follow-up cost					
Years 2, 4, 6, 8, 10					
Battery (p13)	30.4 €	30%	9 €	21 €	Retail price**
Years 3 & 7:					
ENT specialist consultation with audiology assessment	53.1 €	70%	37 €	16 €	CDQP002
Battery (p13)	30.4 €	30%	9 €	21 €	Retail price
Year 3 & 7 total:	**83.5€**		**46 €**	**37 €**	
Years 5 & 9:					
Hearing aid replacement appointment (ENT specialist or GP)	25.0 €	70%	17 €	9 €	C+MMG
Unilateral hearing aid replacement total (years 5 & 9)					
Class I device	**1156.0€**		**426 €**	**730 €**	
Class II device	**1706.0€**		**426 €**	**1 280 €**	
Class III device	**2506.0€**		**426 €**	**2 080 €**	

Legend*: *Based on a survey of audiologists (the prices of class II and class III devices are set by audiologists). ** 5€ for 6 batteries, based on a survey of hearing aid retailers. Batteries have to be changed every 4–20 days, depending on the device.*

Abbreviations*: PuHI, public health insurance; PrHI, private health insurance; DRG, diagnosis-related group; ENT, ear, nose, and throat; CT, computerised tomography; GP, General practioner.*

© 2022 Bonnafous S et al. *JAMA Network Open.*

eFigure 1. Tornado Diagram for Mean Cost per Patient

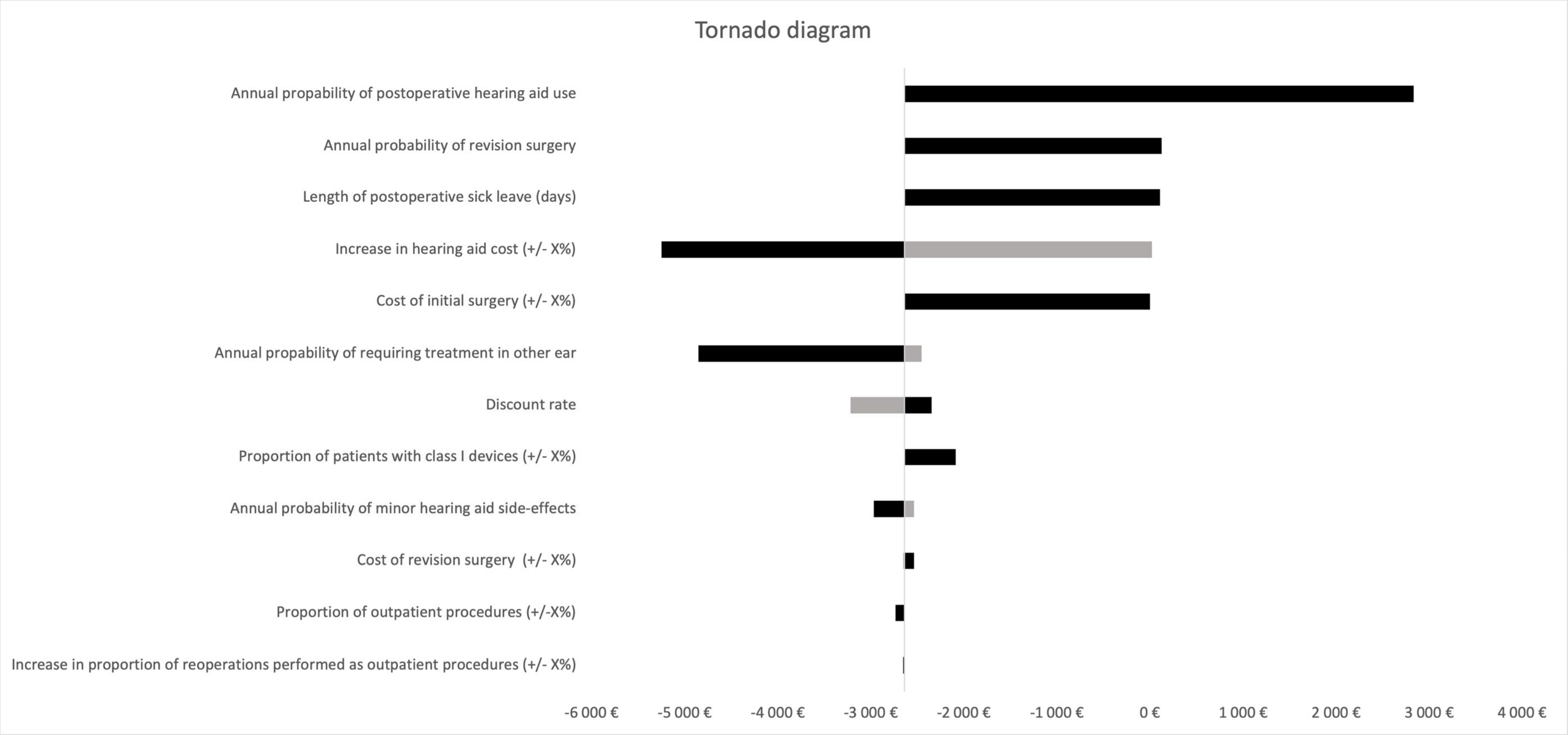

***Legend**: The horizonal axis represents the difference in costs (surgery – hearing aids; positive values mean that surgery is more expensive than hearing aids) for the listed variables. The black (grey) bars represent the differences obtained when lower (upper) estimates are used.*

© 2022 Bonnafous S et al. *JAMA Network Open.*

eTable 3. Lower and Upper Estimates for the Variables Considered in the Sensitivity Analysis

Variable	Lower estimate	Upper estimate
Proportion of outpatient procedures (+/- X%)	0%	66%
Cost of initial surgery (+/- X%)	0%	91%
Increase in the proportion of revision surgery performed as outpatient procedures (+/- X%)	0%	75%
Cost of revision surgery (+/- X%)	10%	91%
Annual probability of revision surgery	0.004	0.1
Length of postoperative sick leave (days)	0.00	16.00
Proportion of patients using class I devices (+/- X%)	0%	100%
Increase in hearing aid cost (+/- X%)	51%	50%
Annual probability of postoperative hearing aid use	0.014	1
Annual probability of minor hearing aid side-effects	0	1
Annual probability of requiring treatment in the other ear	0	0.5
Discount rate	0%	4%

© 2022 Bonnafous S et al. *JAMA Network Open.*
